# Risk stratification of ST-segment elevation myocardial infarction (STEMI) patients using machine learning based on lipid profiles

**DOI:** 10.1186/s12944-021-01475-z

**Published:** 2021-05-06

**Authors:** Yuzhou Xue, Jian Shen, Weifeng Hong, Wei Zhou, Zhenxian Xiang, Yuansong Zhu, Chuiguo Huang, Suxin Luo

**Affiliations:** 1 Department of Cardiology, The First Affiliated Hospital of Chongqing Medical University, NO.1 Youyi Road, Yuzhong District, Chongqing, 400016 China; 2 Department of Medical Imaging, The First Affiliated Hospital of Guangdong Pharmaceutical University, Guangzhou, China; 3 Department of Medicine and Therapeutics, The Chinese University of Hong Kong, Hong Kong, China

**Keywords:** Machine learning, Lipoprotein, ST-segment elevation myocardial infarction, Prognosis, Cardiovascular statin-modified risk

## Abstract

**Background:**

Numerous studies have revealed the relationship between lipid expression and increased cardiovascular risk in ST-segment elevation myocardial infarction (STEMI) patients. Nevertheless, few investigations have focused on the risk stratification of STEMI patients using machine learning algorithms.

**Methods:**

A total of 1355 STEMI patients who underwent percutaneous coronary intervention were enrolled in this study during 2015–2018. Unsupervised machine learning (consensus clustering) was applied to the present cohort to classify patients into different lipid expression phenogroups, without the guidance of clinical outcomes. Kaplan-Meier curves were implemented to show prognosis during a 904-day median follow-up (interquartile range: 587–1316). In the adjusted Cox model, the association of cluster membership with all adverse events including all-cause mortality, all-cause rehospitalization, and cardiac rehospitalization was evaluated.

**Results:**

All patients were classified into three phenogroups, 1, 2, and 3. Patients in phenogroup 1 with the highest Lp(a) and the lowest HDL-C and apoA1 were recognized as the statin-modified cardiovascular risk group. Patients in phenogroup 2 had the highest HDL-C and apoA1 and the lowest TG, TC, LDL-C and apoB. Conversely, patients in phenogroup 3 had the highest TG, TC, LDL-C and apoB and the lowest Lp(a). Additionally, phenogroup 1 had the worst prognosis. Furthermore, a multivariate Cox analysis revealed that patients in phenogroup 1 were at significantly higher risk for all adverse outcomes.

**Conclusion:**

Machine learning-based cluster analysis indicated that STEMI patients with increased concentrations of Lp(a) and decreased concentrations of HDL-C and apoA1 are likely to have adverse clinical outcomes due to statin-modified cardiovascular risks.

**Trial registration:**

ChiCTR1900028516 (http://www.chictr.org.cn/index.aspx).

**Supplementary Information:**

The online version contains supplementary material available at 10.1186/s12944-021-01475-z.

## Background

Dyslipidemia has been considered as a risk factor in atherosclerotic progression [1]. Plasma lipoproteins, including cholesterol esters, apolipoproteins, and triglycerides, can predict adverse outcomes in patients with coronary artery disease (CAD) [2–5]. Nevertheless, the complex and joint relationship of plasma lipoproteins that may interact in a physiological or pathophysiological manner can complicate the analysis and integration in clinical settings [6, 7]. Moreover, none of these lipoproteins could be identified with the “one-size-fits-all” marker of CAD prognosis.

ST-segment elevation myocardial infarction (STEMI) has been recognized as the most acute manifestation of CAD. Although the prognosis of patients with STEMI has improved with the implementation of reperfusion and lipid-lowering strategies, hospitalization and 1-year mortality rates are still at 5–6% and 7–18%, respectively [8]. Statins, well-recognized recommendations for universal use of evidence-based drugs, mainly decrease levels of low-density lipoprotein cholesterol (LDL-C) and long-term mortality [9, 10]. Recent evidence demonstrated that lipid alterations beyond LDL-C are also associated with cardiovascular risk [11]. Several emerging medications displaying direct effects on lipoproteins other than LDL-C have also been investigated [12]. However, other components of the lipid profile as a potentially important part of the overall absolute STEMI related risk assessment have not been fully evaluated. Hence, it is crucial to develop novel strategies for identifying high-risk STEMI subgroups considering lipid profiles.

Unsupervised clustering algorithm, which is an agnostic approach, can segregate patients with similar phenotype without the guidance of an a priori classification system [13]. Previous studies have utilized unsupervised cluster analysis to divide patients with heart failure, pulmonary artery disease, and CAD [14–16]. However, nearly no studies have focused on unsupervised clustering in STEMI patients. Accordingly, this study aimed to generate lipid-derived phenogroups using an unsupervised machine learning method to identify high risk patients with STEMI during follow-up.

## Methods

### Study population and design

In this study, patients diagnosed with STEMI were consecutively enrolled in the First Affiliated Hospital of Chongqing Medical University between December 2014 and December 2018. All participants had STEMI defined by (1) typical chest pain or equal symptoms persisting for more than 30 min, (2) continuous ST-segment elevation in at least two contiguous leads or new left bundle-branch block on an electrocardiogram, and (3) elevated levels of a myocardial enzyme more than twice the upper limit value. Patients were excluded if they were admitted for more than 24 h since symptom’s onset, had missing data, and did not receive primary percutaneous coronary intervention (PCI). Ultimately, 1355 patients had been involved in this study. After admission, the patients were administered medication in adherence to the guideline for STEMI therapy [17]. Written informed consent was provided by all participants, and the study was executed according to the Declaration of Helsinki.

In this cohort, the lipid-associated phenotyping approach entailed (i) an unsupervised consensus clustering analysis to identify the STEMI phenogroups without the constraint of a priori clinical data, (ii) a comparison of clinical characteristics among the lipid-derived clusters, and (iii) a multivariate Cox analysis to validate the association of STEMI phenogroups with all adverse events during follow-up.

### Data collection

Two physicians independently collected the demographic, clinical, laboratory, angiographic, and medication characteristics of the STEMI patients through the hospital record system. The Gensini score, which indicates atherosclerotic plaque burden, was calculated through angiography before the PCI [18, 19]. The post-procedural thrombolysis in myocardial infarction grade was defined according to the operative record files.

Overnight fasting venous blood specimens were obtained for lipid profiles in 24 h of symptom onset. The levels of lipid panels were calculated with a Cobas c701 biochemistry analyzer from Roche Diagnostics (Basel, Switzerland). The following seven candidate lipoprotein variables displaying a strong cardiovascular risk association were chosen for further unsupervised clustering analysis:

1. Total cholesterol (TC)
2. Total triglyceride (TG)
3. High density lipoprotein cholesterol (HDL-C)
4. Low density lipoprotein cholesterol (LDL-C)
5. Apolipoprotein A1 (apoA1)
6. Apolipoprotein B (apoB)
7. Lipoprotein (a) [Lp(a)]

All participants included in the study were regularly contacted (typically every 3 months) via telephone interviews and office visits. The endpoints after discharge were defined as all-cause mortality, and all-cause and cardiac rehospitalization events. With a 904-day median follow-up (interquartile range: 587–1316), 166 deaths were ultimately registered. All follow-up activities were ended on May 1, 2020.

### Unsupervised machine learning clustering analysis

The normality of the distribution of the seven lipoprotein variables was first assessed. Lp(a) was converted as Ln [Lp(a)] given a shewed distribution, and then the log-transformed variable was applied to the subsequent analysis. Thereafter, the seven variables (TC, TG, HDL-C, LDL-C, apoA1, apoB, and Ln [Lp(a)]) were Z-score transformed (to a mean of 0 and variance of 1) to minimize the effect of variables with a larger variance on clustering. Then an unsupervised consensus clustering was implemented to sort STEMI patients into phenogroups based on the lipid profile using the “ConsensusClusterPlus” package in R [20]. Consensus clustering with 1000 resampling iterations (80% of patients/subsample) among a cluster number (*k*) range of *k* = 2–20 was utilized. The *k* optimal clustering stability was verified through the proportion of ambiguously clustered pairs (PAC) and consensus matrix heatmaps [20, 21]. Four algorithms (namely, the k-means, hierarchical, partitioning around medoids, and k-medoids algorithms) with seven different distance metrics (28 total combinations) were applied to determine the input parameters of clusters with the best internal validity through the “fpc” package. Ultimately, the k-medoids algorithm and Pearson distance were applied for consensus clustering, and *k* = 3 was chosen as the best optimal number of clusters through PAC and consensus heatmap (Supplementary Table 1, and Supplementary Figure 1).

### Principal component analysis (PCA)

To identify the discriminative performance of the unsupervised machine learning algorithm, principal component analysis (PCA) was applied as a dimensional reduction technique to summarize the overall clinical variation of the lipid profiles. The first three principal components (PCs) (accounting for more than 80% variance) were selected for further analysis (Supplementary Figure 2A). Differences in PC1, PC2, and PC3 among the three phenogroups were also identified (Supplementary Figure 2B-D). Finally, patients were mapped into a coordinate system based on the first three PCs (Supplementary Figure 3).

### Clinical comparison of phenogroups

Differences in demographic, clinical, laboratory, angiographic and medication characteristics among the lipid-derived phenogroups were compared. Continuous variables were summarized as mean (SD) or median (interquartile range) depend on their normal or non-normal distribution, correspondingly, whereas categorical variables were summarized as frequencies (percentage). To examine the differences among the phenogroups, one-way analysis of variance and Kruskal-Wallis test were conducted on the normally and non-normally distributed data. A Chi-squared test was used for categorical variables.

Next, the all-cause mortality approximations obtained with the Kaplan-Meier curves were compared across different phenogroups through a log-rank test. Patients in different phenogroups who were re-admitted to the hospital due to all-cause or cardiac events (including re-myocardial infarction, heart failure, cardiogenic shock, arrhythmia, major bleeding, and cardiac mortality) were also compared via Kaplan-Meier curves.

Multivariate Cox proportional hazards regression was implemented to explore the association of the phenogroups with all adverse outcomes. Multivariable models were adjusted for age, gender, history of diabetes, hypertension, smoking status, culprit artery, creatinine, left ventricular ejection fraction, high-sensitivity C-reactive protein, cardiac troponin I, time to balloon (h), and thrombolysis in myocardial infarction grade (≤II/III). All statistical analyses were conducted with R version 3.6.3 (R Foundation for Statistical Computing, Vienna, Austria). For these analyses, *P*value ≤ 0.05 was considered as statistically significant.

## Results

### Machine learning-based lipid-derived phenogroups

**Fig. 1:**
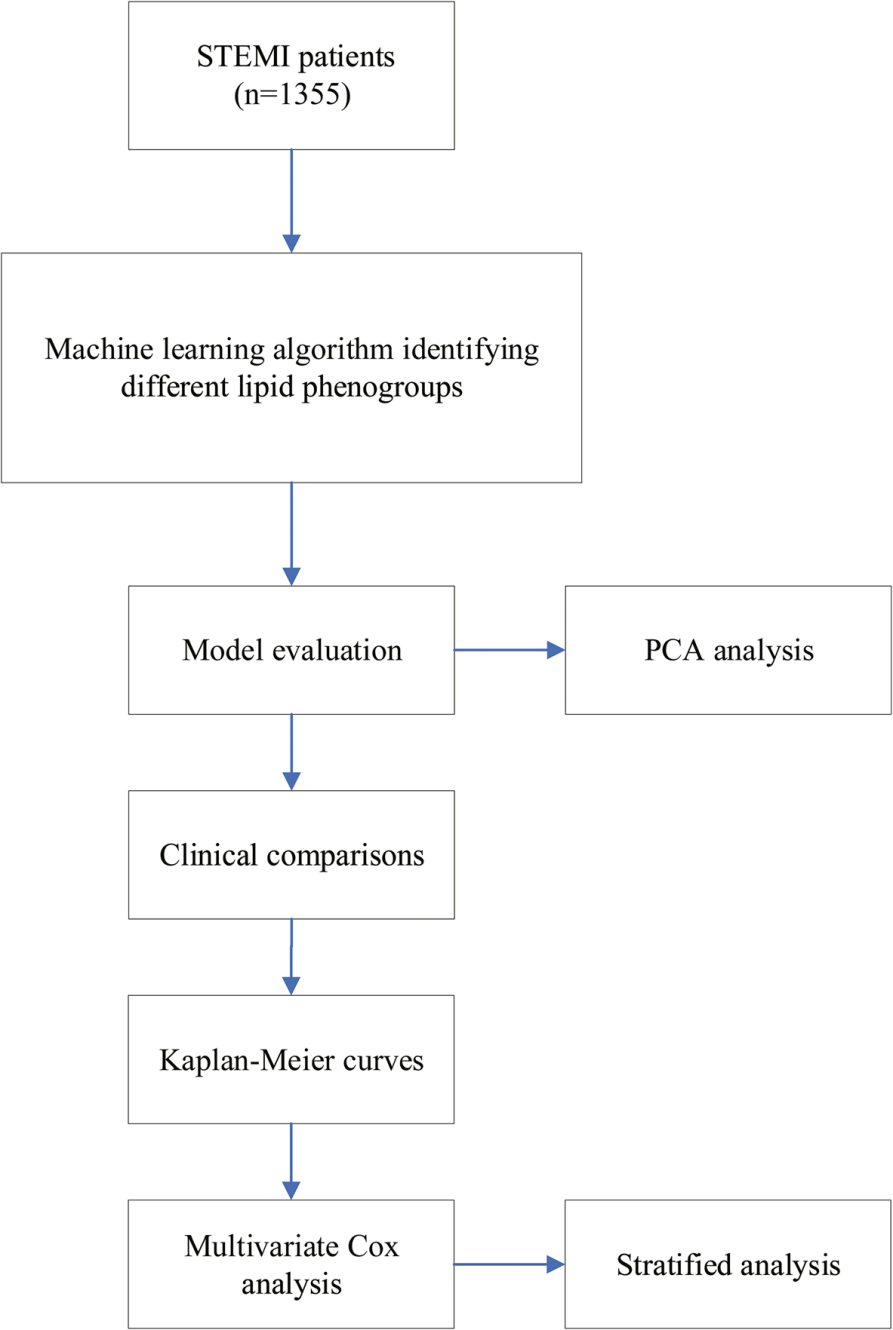
Overall study design. STEMI, ST-segment elevation myocardial infarction

**Fig. 2:**
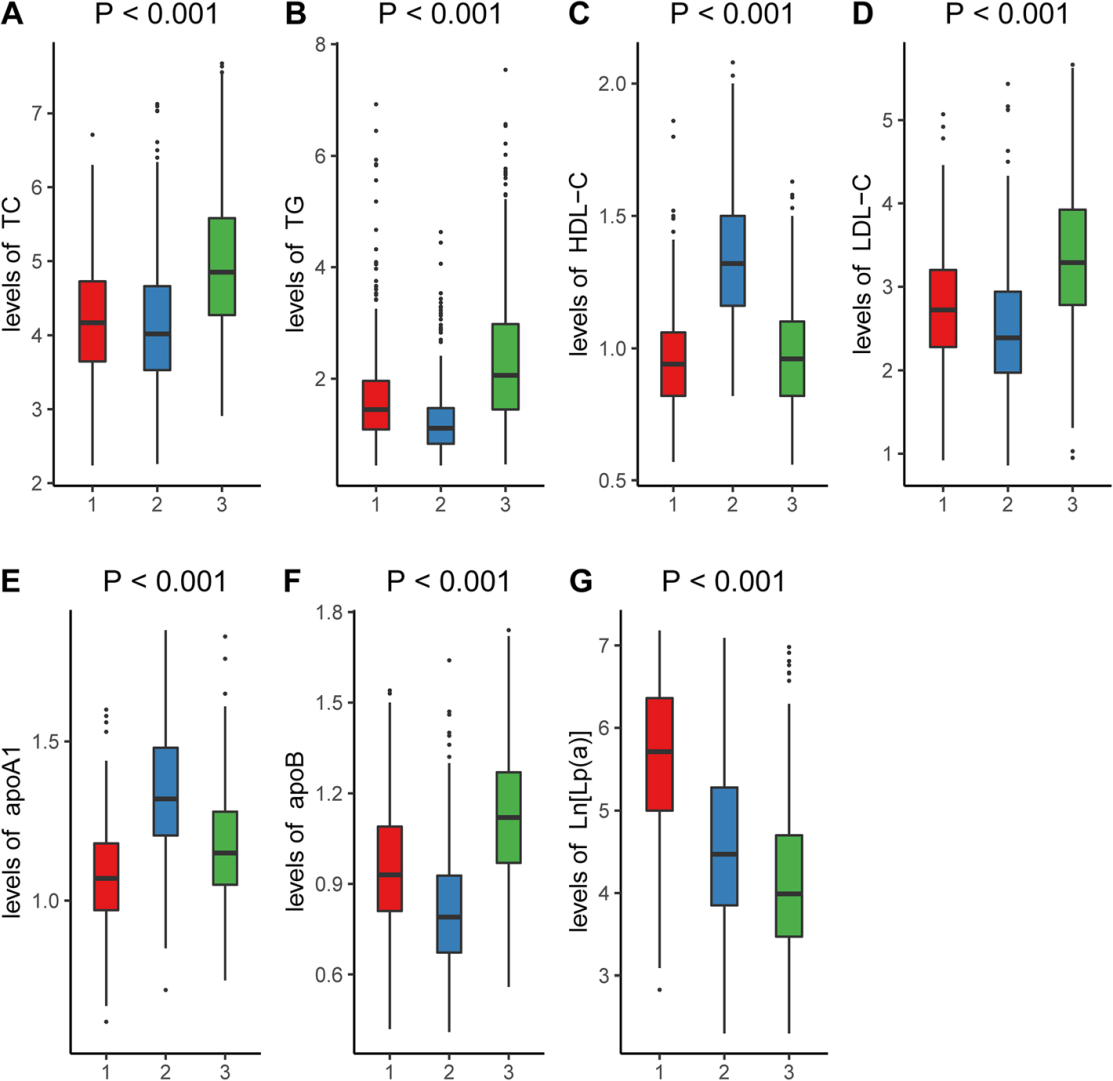
Lipid profile levels among three phenogroups

Figure 1 shows the overall design of the study. The three phenogroups that displayed a distinct lipid profile pattern were identified using an unsupervised machine learning algorithm. The lipid profile levels for the different phenogroups are illustrated in Fig. 2. Patients in phenogroup 1 had the lowest concentrations of apoA1 and HDL-C and moderate levels of TC, TG, LDL-C, and apoB, whereas those in phenogroup 2 had the lowest concentrations of TC, TG, LDL-C, and apoB and the highest levels of apoA1 and HDL-C. Conversely, patients in phenogroup 3 had the highest levels of TC, TG, LDL-C, and apoB and intermediate levels of HDL-C and apoA1. Lp(a) decreased from phenogroup 1 to 3. All seven lipoprotein variables were significantly different among the three phenogroups (*P* < 0.001, Table 1).

**Table 1:** The levels of lipid profiles across different lipid-derived phenogroups

Variables	Cluster 1 (*n* = 415)	Cluster 2 (*n* = 496)	Cluster 3 (*n* = 444)	*P* value
TC, mmol/L	4.15 (0.84)	4.11 (0.92)	5.05 (1.17)	< 0.001
TG, mmol/L	1.70 (1.17)	1.25 (0.77)	2.59 (2.24)	< 0.001
HDL-C, mmol/L	0.94 (0.20)	1.37 (0.30)	0.97 (0.22)	< 0.001
LDL-C, mmol/L	2.72 (0.73)	2.47 (0.76)	3.41 (1.01)	< 0.001
apoA1, g/L	1.05 (0.21)	1.36 (0.22)	1.16 (0.19)	< 0.001
apoB, g/L	0.95 (0.21)	0.80 (0.22)	1.16 (0.37)	< 0.001
Lp(a), mg/L	322 (150–602)	87 (46.5–200.5)	54 (31–109)	< 0.001

*Abbreviations*: *TC* total cholesterol, *TG* total triglyceride, *HDL-C* high-density-lipoprotein cholesterol, *LDL-C* low-density-lipoprotein cholesterol, *apo* apolipoprotein, *Lp(a)* lipoprotein (a)

**Fig. 3:**
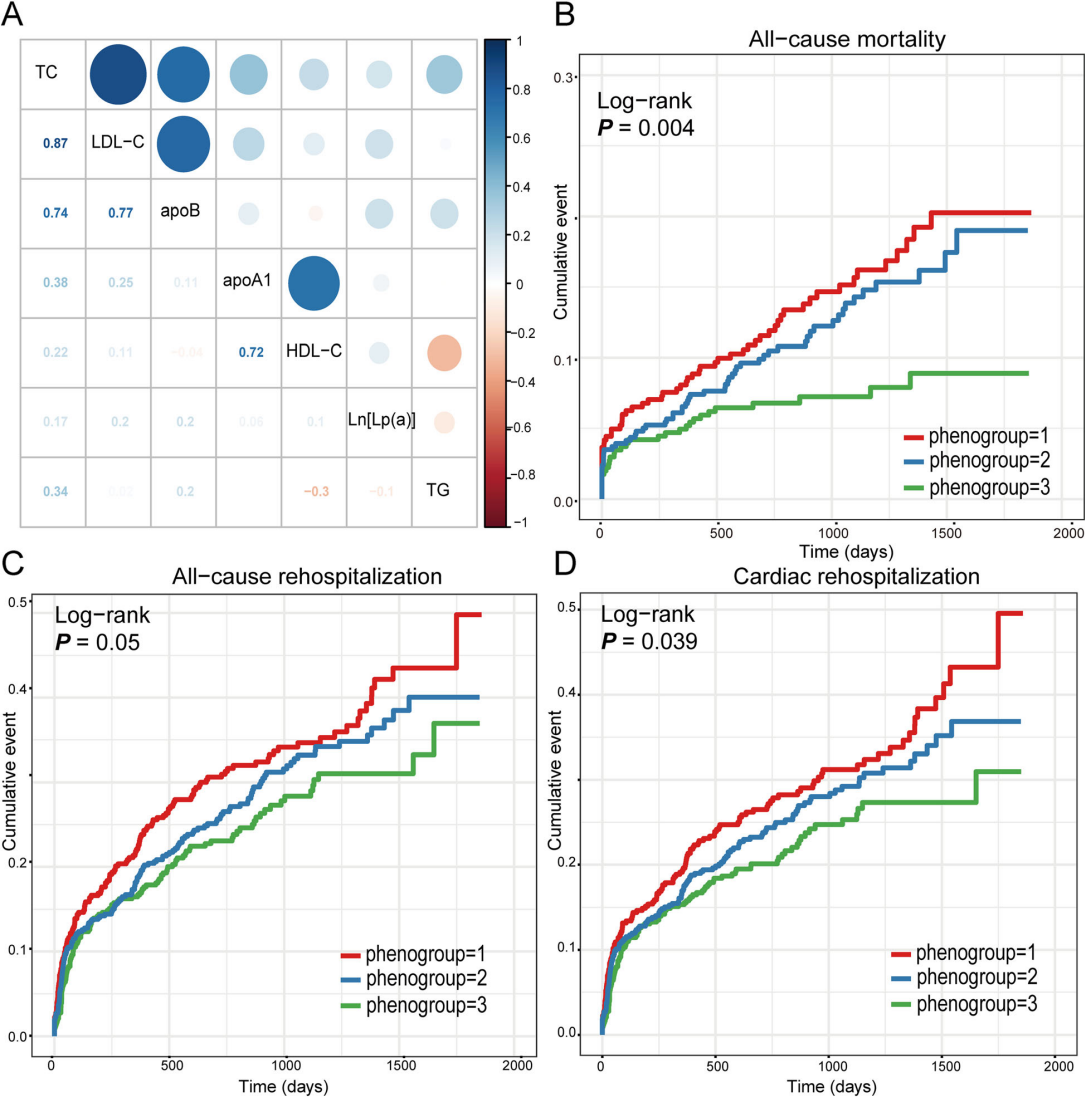
Clinical outcomes among different phenogroups during follow-up. **a** Intercorrelation among different lipoprotein variables; Kaplan-Meier survival curves of **b** all-cause mortality; **c** all-cause rehospitalization; and **d** cardiac rehospitalization among different phenogroups

Pearson correlations among the seven lipoprotein variables were conducted (Fig. 3a). There were moderately strong positive associations (*r* > 0.5) among TC, LDL-C, and apoB and between apoA1 and HDL-C. The other correlations demonstrated either a weak positive or a negative correlation.

### Baseline characteristics among phenogroups

The baseline characteristics, including demographics, clinical signs, angiographic findings and medications are presented in Table 2. Patients in phenogroup 2 were older (*P* < 0.001) and had the lowest percentage of men (*P* < 0.001), dyslipidemia (*P* < 0.001), and smoking status (*P* < 0.001). Further, they had the highest percentage of left anterior descending artery occlusion (*P* = 0.02). Patients in phenogroup 2 had the lowest hemoglobin A1c (*P* < 0.001) but the highest hemoglobin (*P* < 0.001) and free thyroxine (*P* = 0.002). Patients in phenogroup 1 had the highest creatinine (*P* < 0.001) levels. Serum free triiodothyronine levels were lower in phenogroup 1 and 2 than in phenogroup 3 (*P* = 0.004). No significant difference in statin use (*P* = 0.56) was detected among the phenogroups, but aspirin (*P* = 0.003) and beta-blocker (*P* = 0.024) use were the highest, and diuretic (*P* = 0.004) use was the lowest in phenogroup 3.

**Table 2:** Baseline demographic and clinical characteristics of the study participants in the across lipid-derived phenogroups

Variables	Phenogroup 1 (*n* = 415)	Phenogroup 2 (*n* = 496)	Phenogroup 3 (*n* = 444)	*P* value
Age, years	63.3 (12.5)	67.1 (11.6)	59.5 (12.5)	< 0.001
Male, sex	349 (84.1%)	350 (70.6%)	377 (84.9%)	< 0.001
Previous event				
Myocardial infarction	19 (4.6%)	19 (3.8%)	18 (4.1%)	0.84
PCI	13 (3.1%)	17 (3.4%)	9 (2.0%)	0.42
Diabetes	91 (21.9%)	90 (18.1%)	108 (24.3%)	0.061
Hypertension	219 (52.8%)	262 (52.8%)	219 (49.3%)	0.49
Dyslipidemia	46 (11.1%)	30 (6.0%)	64 (14.4%)	< 0.001
Chronic kidney disease	19 (4.6%)	11 (2.2%)	10 (2.3%)	0.084
Smoking status	301 (72.5%)	293 (59.1%)	327 (73.6%)	< 0.001
Time to balloon, h	6.0 (4.0–11.0)	6.0 (3.5–11.0)	5.0 (3.0–9.0)	0.062
SBP, mmHg	123.3 (25.5)	124.9 (26.3)	127.0 (23.7)	0.092
HR, bpm	81.9 (19.2)	81.0 (19.3)	83.2 (16.8)	0.18
BMI, Kg/m2	24.0 (3.5)	23.0 (3.7)	25.1 (3.4)	< 0.001
Culprit artery				0.02
LAD	183 (44.1%)	278 (56.0%)	227 (51.1%)	
LCX	44 (10.6%)	62 (8.1%)	47 (10.6%)	
RCA	178 (42.9%)	169 (34.1%)	157 (35.4%)	
LM	4 (1.0%)	1 (0.2%)	3 (0.7%)	
Multivessel	6 (1.4%)	8 (1.6%)	10 (2.2%)	
Gensini score	56.3 (23.7)	53.3 (25.2)	55.2 (26.1)	0.20
Killip class (> II)	116 (28.0%)	142 (28.6%)	100 (22.5%)	0.092
Postprocedural TIMI (< II)	29 (7.0%)	33 (6.7%)	25 (5.6%)	0.71
Hb, g/L	134.6 (19.2)	141.5 (20.7)	137.9 (20.2)	< 0.001
HbA1c, %	6.6 (1.7)	6.4 (1.6)	6.9 (1.7)	< 0.001
Creatinine, μmol/L	96.6 (33.8)	81.8 (42.7)	81.0 (38.8)	< 0.001
FT3, pg/ml	2.87 (0.51)	2.87 (0.94)	2.98 (0.68)	0.004
FT4, ng/dl	0.92 (0.21)	0.95 (0.39)	0.89 (0.16)	0.002
hsTSH, μIU/ml	0.92 (0.57–1.7)	0.98 (0.54–1.68)	0.98 (0.53–1.57)	0.99
cTNI, ug/L	98.4 (9.8–473.3)	74.7 (6.9–462.8)	90.1 (9.1–471.0)	0.35
NT-proBNP	0.96 (0.34–186)	1.33 (0.34–169)	0.70 (0.27–162)	0.16
LVEF, %	55.5 (7.1)	54.3 (8.1)	55.3 (7.4)	0.059
Aspirin	406 (97.8%)	489 (98.6%)	444 (100.0%)	0.003
Statin	415 (100.0%)	494 (99.6%)	442 (99.5%)	0.56
Beta-blocker	336 (81.0%)	403 (81.3%)	386 (86.9%)	0.024
Diuretics	180 (43.4%)	219 (44.2%)	153 (34.5%)	0.004

*Abbreviations*: *CABG* coronary artery bypass grafting, *PCI* percutaneous coronary intervention, *SBP* systolic blood pressure, *HR* heart rate, *BMI* body mass index, *LAD* left anterior descending artery, *LCX* left circumflex artery, *RCA* right coronary artery, *LM* left main artery, *TIMI* thrombolysis in myocardial infarction, *Hb* hemoglobin, *HbA1c* hemoglobin A1c, *FT3* free triiodothyronine, *FT4* free thyroxine, *hsTSH* high-sensitive thyroid-stimulating hormone, *cTNI* cardiac troponin I, *NT-proBNP* N-terminal pro b-type natriuretic peptide, *LVEF* left ventricular ejection fraction

### Association between the phenogroups and prognosis

During follow up, 166 deaths were registered. The incidence of all-cause mortality was significantly higher in phenogroup 1 than in phenogroups 2 and 3 (7.9% vs. 15.4% vs. 13.7%, *P* = 0.004 respectively) (Fig. 3b). In addition, 422 all-cause and 378 cardiac rehospitalization events were recorded. All-cause (40.7% vs. 36.6% vs. 29.1%, *P* = 0.05) and cardiac (37.4% vs. 32.9% vs. 25.0%, *P* = 0.039) rehospitalizations were more frequent among patients in phenogroup 1 than those in phenogroups 2 and 3. In general patients in phenogroup 3 were associated with better prognosis (Fig. 3c and d).

In multivariable adjusted Cox models, phenogroup 2 (vs. phenogroup 1) was found to be closely related to a decreased possibility of all-cause mortality [hazard ratio (HR) = 0.62, 95% confidence interval (CI) (0.37–0.99)], all-cause rehospitalization [HR = 0.80, 95% CI (0.61–0.98)], and cardiac rehospitalization [HR = 0.75, 95% CI (0.57–0.96)] (Table 3). Furthermore, patients in phenogroup 3 (vs. phenogroup 1) also had a lower risk of all-cause mortality (HR 0.54, 95% CI 0.28–0.96), all-cause rehospitalization (HR 0.76, 95% CI 0.57–0.97), and cardiac rehospitalization (HR 0.74, 95% CI 0.54–0.97) (Table 3).

**Table 3:** Multivariable cox analysis of association between lipid-derived phenogroups and outcomes during follow-up

	Phenogroup 1 (*n* = 415)		Phenogroup 2 (*n* = 496)			Phenogroup 3 (*n* = 444)		
Outcomes	Events	Reference	Events	HR (95% CI)	*P*-value	Events	HR (95% CI)	*P*-value
All-cause mortality	64 (15.4%)	Ref.	67 (13.7%)	0.62 (0.37–0.99)	0.04	35 (7.9%)	0.54 (0.28–0.96)	0.001
All-cause rehospitalization	149 (35.9%)	Ref.	160 (32.3%)	0.80 (0.61–0.98)	0.03	113 (25.5%)	0.76 (0.57–0.97)	0.01
Cardiac rehospitalization	137 (33.0%)	Ref.	144 (29.0%)	0.75 (0.57–0.96)	0.02	97 (21.8%)	0.74 (0.54–0.97)	0.04

Outcomes were adjusted for age, gender, history of diabetes, hypertension, smoking status, culprit artery, creatinine, LVEF, hsCRP, cTNI, time to balloon (h), post procedural TIMI (≤2)

*Abbreviations*: *LVEF* left ventricular ejection fraction, *hsCRP* high sensitivity C-reactive protein, *cTNI* cardiac troponin I, *TIMI* thrombolysis in myocardial infarction

**Fig. 4:**
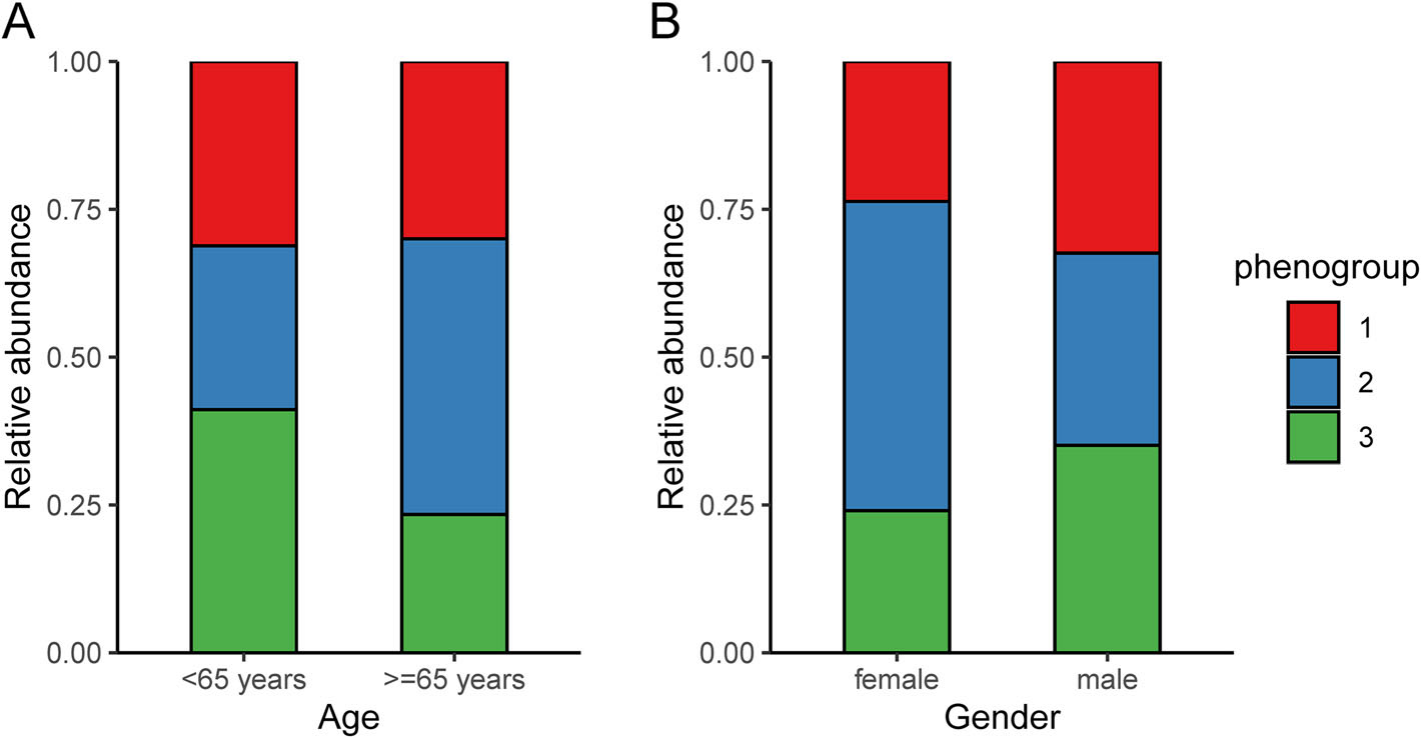
Bar chart of relative frequency of phenogroups for different (**a**) age and (**b**) gender subgroups

The proportions of different phenogroups stratified by age (< 65 years or ≥ 65 years) and gender (male or female) are presented in Fig. 4. The percentage of phenogroup 1 in young (< 65 years) and old (≥65 years) group was similar (31.2% vs. 30.0%). However, the percentage of phenogroup 2 in young group was less than that in old group (27.7% vs. 46.6%). Furthermore, the percentage of phenogroup 2 (52.3% vs. 32.5%) was decreased but percentages of phenogroup 1 (23.7% vs. 32.4%) and 3 (24.0% vs. 35.0%) were increased from female to male group.

## Discussion

In this first unsupervised machine learning-based clustering study of STEMI patients, three distinct phenogroups were identified according to multiple serum lipoproteins levels, revealing different lipoprotein expression patterns, and baseline characteristics. Patients in phenogroup 1 with the highest Lp(a) and lowest apoA1 and HDL-C had the worst prognosis in the adjusted Cox analysis.

STEMI, which is one of the most critical clinical situations of CAD, is caused by plaque rupture or erosion with a thrombus obstruction of the epicardial coronary artery and then transmural ischemia [22]. Despite the substantial improvement of prognosis among STEMI patients due to the development of reperfusion and preventive measures over several decades [23], STEMI remains the leading cause of mortality and morbidity globally [24, 25]. Szummer et al. [26] reported that the first-year mortality of STEMI patients in Sweden remains at 14.1%, even with the wide implementation of a variety of treatment strategies, including PCI, use of statin and beta-blocker, dual antiplatelet therapy, and implementation of angiotensin-converting enzyme inhibitor/angiotensin-receptor blocker. Hence, improvement in the risk stratification of STEMI patients is necessary for further improvement to prognoses.

Machine learning algorithms can identify an underlying pattern in complicated and various data. Furthermore, unsupervised clustering analysis can shed light on the non-linear interactions among variables without a priori attention to clinical events [13]. Recently, machine learning based approaches have been implemented to stratify patients with heart failure based on echocardiographic parameters [27–29]. Additionally, machine learning analyses have been used to phenomap prognostic categories and discover the responders of cardiac resynchronization therapy among heart failure patients through mixed-data phenotypic variables [14, 30]. However, no studies have focused on recognizing the different patterns of lipoprotein expression through unsupervised consensus clustering in STEMI patients; Moreover, all lipoprotein variables included in this study were associated with cardiovascular risk. Hence, investigating the lipoprotein expressed features in phenogroups with poor prognosis could be helpful for risk stratification.

Unsupervised clustering algorithm is an information-driven method to analyze the intrinsic relationship of high-dimensional data and then identify the existence of specific subtype of patients [20]. This method is also helpful in exploring the complicated lipoprotein variables. Furthermore, this analysis is focused on extracting valuable insights from the dataset, not associating with clinical outcomes. Hence, this method provides an open-ended exploratory perspective on the data and can identify new lipoprotein phenogroups [31].

Surprisingly, phenogroup 3 with the highest levels of LDL-C, TC, apoB, and TG was associated with the best prognosis, whereas phenogroup 2 with the lowest levels of LDL-C, TC, TG, and apoB and highest levels of HDL-C and apoA1 had relatively increased risk for adverse clinical outcomes. The reason for this result is that phenogroup 2 comprised much older patients and a larger percentage of female patients compared to the other phenogroups (in phenogroup 2, 29.4% of patients were female and 59.9% were ≥ 65 years old). Recent findings support that female STEMI patients have enhanced risk of death compared to male STEMI [32]. Furthermore, patients in phenogroups 2 and 3 showed a similar risk of clinical outcomes after discharge in the multivariate Cox analysis, which indicates that higher LDL-C increases the risk in younger phenogroups. On the contrary, patients in phenogroup 1 with the highest Lp(a) and lowest apoA1 and HDL-C levels had the worst clinical outcomes even after the differences in age and gender were adjusted. Hence, the lipoprotein characteristics of phenogroup 1 must be identified.

After years of lipid-lowering therapy development, statins have been widely used, especially in STEMI patients. The Statins Evaluation in Coronary Procedures and Revascularization (SECURE-PCI) study revealed that statin therapy during hospitalization brought significant benefits for STEMI patients undergoing PCI [33]. Statin primarily acts on LDL-C and high-intensity statin therapy is predicted to decrease LDL-C by more than 50% [34]. Furthermore, almost every patient included into the study was treated with a statin and this standard post-STEMI treatment was equally distributed across different phenogroups. However, residual cardiovascular risk continues to be high, despite statin therapy [35]. Lipoprotein variables, including HDL-C, apoA1 and Lp(a), have been reported as predictors of statin-modified cardiovascular risk.

Mechanically, the major lipid effect of statins is the lowering of circulating concentrations of LDL-C and TG [36]. However, the influence of statins on HDL-C is minimal [37]. A newly published meta-analysis, which enrolled 20 randomized controlled trials among Asian population, revealed that statin/ezetimibe combination therapy slightly increased HDL-C by 0.02 mmol/L [38]. Furthermore, it has been recently documented that an augmentation in the serum concentration of Lp(a) is associated with statin therapy [39].

ApoA1, the main protein constituent of high-density lipoprotein (HDL) particles, plays a critical role in reverse cholesterol transport, anti-inflammatory, antithrombotic, and antioxidant activities [40]. Furthermore, emerging evidence indicates that HDL and apoA1 are correlated with the improvement of stent biocompatibility after PCI [41]. Other clinical trials identified that lower HDL-C is associated with cardiovascular events in patients with type 2 diabetes mellitus and stable ischemic heart disease even with an optimal control of LDL-C levels [42, 43]. In contrast, the unexpected ratio of relatively high prognostic risk to patients with higher levels of HDL-C and apoA1 in phenogroup 2 in this study may be due to older age and a larger percentage of female patients than those in phenogroup 3.

Lp(a) consists of LDL-like particles containing apoB-100 and its covalently linked glycoprotein apo(a) particle, which is determined by the LPA gene [44]. A Mendelian randomization study demonstrated that elevated Lp(a) is a strong and causal risk factor of atherosclerotic cardiovascular disease [45]. Traditional lipid-lowering therapies including statins, fibrates, and ezetimibe inefficiently lower Lp(a) levels [46]. New and emerging medicines such as proprotein convertase subtilisin/kexin type 9 (PCSK9) inhibitors and antisense oligonucleotides targeting apolipoprotein(a) (IONIS-APO(a) Rx and IONIS-APO(a)-L Rx) could reduce Lp(a) by 30–40 and 60%–70%, respectively [47–50]. Recent guidelines recommend that patients with extremely high Lp(a) levels should be treated with PCSK9 inhibitors instead of statin [9]. However, more evidence related to the clinical application of new Lp(a)-lowering drugs in STEMI patients is lacking and nearly none of the patients in the present study had received PCSK9 inhibitor therapy. Additionally, patients in phenogroups 1 and 3 could be classified into type IIa and type IIb according to Fredrickson classification, respectively [51, 52]. Homma Y et al. [53] observed that simvastatin did not alter Lp(a) levels in either type IIa or type IIb dyslipidemia. Furthermore, an observational study reported that high concentrations of Lp(a) through low LPA kringl-IV type-2 number of repeats were associated with a high risk of mortality in the general population [54]. Additionally, two prospective trials demonstrated that cardiovascular disease risk associated with elevated Lp(a) remained with LDL-C levels below 2.5 mmol/L [55]. Moreover, individuals who underwent PCI with LDL-C levels below 2.6 mmol/L still had worse all-cause mortality and acute coronary syndrome after their levels of Lp(a) had increased [56].

## Study strengths and limitations

This study is the first to identify the association of different lipoprotein phenogroup with prognosis in STEMI patients through machine learning analysis. More importantly, the relationship between lipid-derived phenogroups and outcomes is still significant after adjusting.

However, limitations of the study should be noticed. First, participants enrolled in this study were patients with STEMI only from one hospital; hence, a prospective and multicenter data may be needed in the future. Second, another independent dataset should be used for validation of the unsupervised clustering analysis. Third, the levels of lipoproteins during follow-up were not collected in this study, which may be important for further stratifying work. Finally, limitations of the follow-up method in this study led to be incapable of exploring the association between lipid-derived phenogroups and cardiac mortality, and the mean follow-up time of 2.5 years was relatively shorter than some other studies.

## Conclusions

The present study identified three phenogroups with different lipoprotein features by using machine learning algorithm in STEMI patients. Patients in phenogroup 1 with the highest Lp(a) but the lowest apoA1 and HDL-C had highest mortality, all-cause and cardiac rehospitalization rates at follow-up. This association remained significant in multivariable adjusted Cox models. Our findings revealed that STEMI patients with high Lp(a), and low HDL-C and apoA1 should be concerned, regardless of age and gender. The administration of Lp(a)-lowering drugs such as PCSK9 inhibitors and antisense oligonucleotides in STEMI patients with high Lp(a) may need to be recommended in the future guidelines.

## Supplementary Information

**Additional file 1: Supplementary Figure 1.** Consensus clustering; measuring consensus and determining the number of clusters (k optimal) (A) heatmap of the consensus matrix for k = 2; (B) heatmap of the consensus matrix for k = 3; (C) heatmap of the consensus matrix for k = 4; (D) empirical cumulative distribution function (CDF) plot *k* values between 2 and 10; (E) Tracking plot of *k* values ranging from 2 to 10.

**Additional file 2: Supplementary Figure 2.** Principal component analysis (PCA) of lipid profiles in ST-segment elevation myocardial infarction (STEMI) patients. (A) Scree plot showing the first seven principal components (PCs) of variation in lipid profiles; Box plots of (B) PC1, (C) PC2, and (D) PC3 among the three phenogroups.

**Additional file 3: Supplementary Figure 3.** Three-dimensional plot of principal component analysis (PCA) results. PC, principal component; red, phenogroup 1; blue, phenogroup 2; green, phenogroup 3.

**Additional file 4: Supplementary Table 1.** Cluster validity statistics for various consensus clustering algorithm-distance metric combinations.

**Additional file 5: Supplementary Table 2.** Consensus clustering: determine the cluster number that optimizes consensus (k optimal) via proportion of ambiguously clustered pairs metric (k-medoids + pearson).

## Data Availability

Data are available for proper requests.

## Abbreviations

95% CI: 95% confidence interval
PAC: Proportion of ambiguously clustered pairs
PCA: Principal component analysis
PC: Principal components
apoB: Apolipoprotein B
CAD: Coronary artery disease
HDL-C: High-density lipoprotein cholesterol
HR: Hazard ratio
LDL-C: Low-density lipoprotein cholesterol
Lp(a): Lipoprotein (a)
PCI: Percutaneous coronary intervention
PCSK9: Proprotein convertase subtilisin/kexin type 9
STEMI: ST-segment elevation myocardial infarction
TC: Total cholesterol
TG: Total glyceride
