# Supplementary figures and images for: Risk stratification of ST-segment elevation myocardial infarction (STEMI) patients using machine learning based on lipid profiles
Source: Lipids Health Dis. 2021 May 6;20:48. doi: 10.1186/s12944-021-01475-z (PMC8101132; doi:10.1186/s12944-021-01475-z)

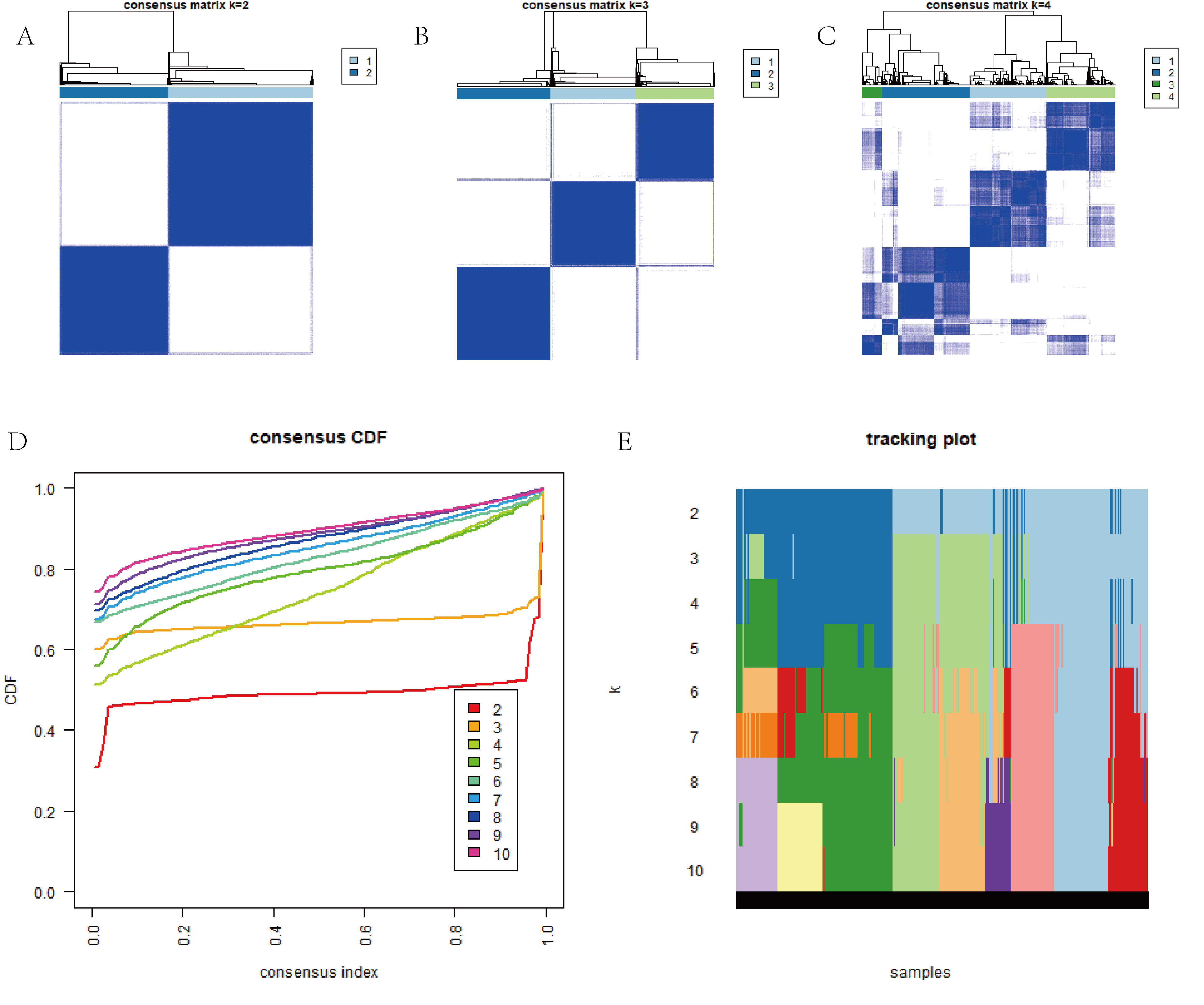

Supplement: Supplementary file 1 — Additional file 1: Supplementary Figure 1. Consensus clustering; measuring consensus and determining the number of clusters (k optimal) (A) heatmap of the consensus matrix for k = 2; (B) heatmap of the consensus matrix for k = 3; (C) heatmap of the consensus matrix for k = 4; (D) empirical cumulative distribution function (CDF) plot k values between 2 and 10; (E) Tracking plot of k values ranging from 2 to 10. [file 12944_2021_1475_MOESM1_ESM.tif]

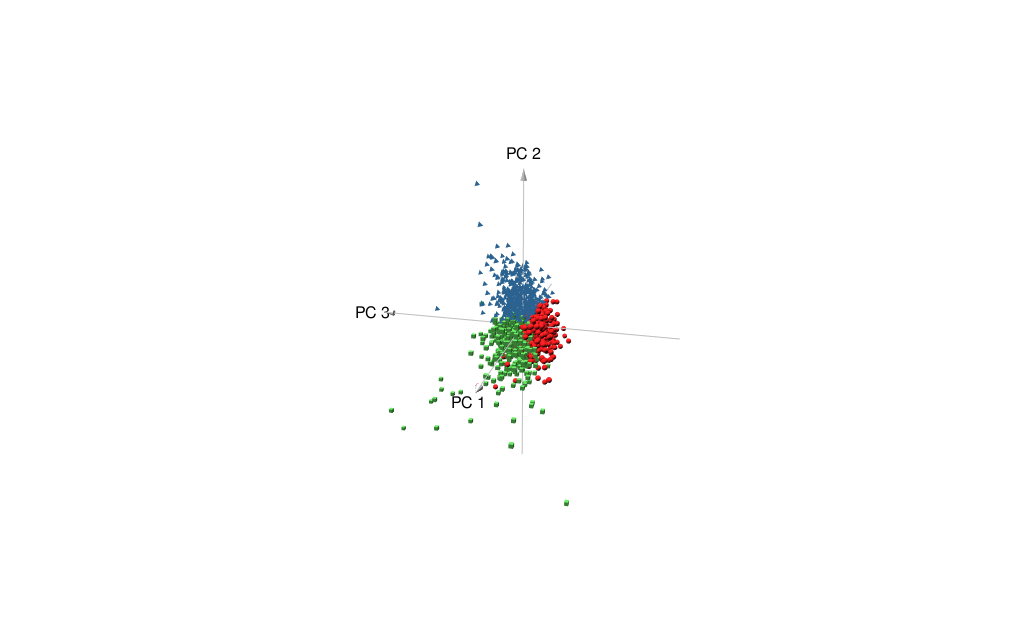

Supplement: Supplementary file 3 — Additional file 3: Supplementary Figure 3. Three-dimensional plot of principal component analysis (PCA) results. PC, principal component; red, phenogroup 1; blue, phenogroup 2; green, phenogroup 3. [file 12944_2021_1475_MOESM3_ESM.png]
