# Supplementary material for: Risk stratification of ST-segment elevation myocardial infarction (STEMI) patients using machine learning based on lipid profiles
Source: Lipids Health Dis. 2021 May 6;20:48. doi: 10.1186/s12944-021-01475-z (PMC8101132; doi:10.1186/s12944-021-01475-z)
Supplement: Supplementary file 5 — Additional file 5: Supplementary Table 2. Consensus clustering: determine the cluster number that optimizes consensus (k optimal) via proportion of ambiguously clustered pairs metric (k-medoids + pearson). [file 12944_2021_1475_MOESM5_ESM.docx]

Supplementary table 2. Consensus clustering: determine the cluster number that optimizes consensus (k optimal) via proportion of ambiguously clustered pairs metric (k-medoids + pearson).

| Cluster number (k) | Proportion of ambiguously clustered pairs (PAC) |
| --- | --- |
| k=2 | 0.044 |
| k=3 | 0.038 |
| k=4 | 0.373 |
| k=5 | 0.293 |
| k=6 | 0.231 |
| k=7 | 0.217 |
| k=8 | 0.219 |
| k=9 | 0.181 |
| k=10 | 0.174 |
